# Innominate artery direct cannulation provides brain protection during total arch replacement for acute type A aortic dissection

**DOI:** 10.1186/s13019-022-01919-2

**Published:** 2022-06-22

**Authors:** Xiang Kong, Peng Ruan, Jiquan Yu, Hui Jiang, Tianshu Chu, Jianjun Ge

**Affiliations:** Department of Cardiovascular Surgery, The First Affiliated Hospital of USTC, Division of Life Sciences and Medicine, University of Science and Technology of China (USTC), Hefei, 230001 Anhui China

**Keywords:** Aortic dissection, Aortic arch, Direct innominate cannulation, Cerebral perfusion

## Abstract

**Background:**

This study aimed to investigate the safety of direct innominate arterial (IA) cannulation using a pediatric arterial cannula to establish selective antegrade cerebral perfusion (ACP) during total arch replacement (TAR) for acute Stanford type A aortic dissection (ATAAD).

**Methods:**

This retrospective study included patients with ATAAD who underwent TAR with the frozen elephant trunk (FET) technique between October 2020 and November 2021. Patients treated with direct IA cannulation using a pediatric arterial cannula for selective anterograde cerebral perfusion were included in the study.

**Results:**

Of the 29 patients, 24 (82.8%) were male. The average age was 50.9 ± 9.47 years. Proximal repair included aortic root plasty (27 patients, [93.1%]) and Bentall surgery (2 patients, [6.9%]). Perioperative mortality and stroke rates were 3.4% and 6.9%, respectively. The mean lowest core temperature was 23.8 ± 0.74 °C and the mean ACP time was 25 ± 6.4 min. The aortic cross-clamp and cardiopulmonary bypass times were 141 ± 28 and 202 ± 29 min, respectively. There were no cases of IA injuries.

**Conclusion:**

Direct IA cannulation using a pediatric arterial cannula is a simple, safe, and effective technique for establishing ACP during TAR with the FET technique for ATAAD and can avoid the potential complications of axillary artery cannulation.

## Background

Nerve injury remains the most dangerous complication of aortic arch surgeries. Current protective strategies to reduce such complications include systemic hypothermia, minimization of circulatory arrest time, and maintanance of cerebral blood perfusion. Although it is debatable whether cerebral perfusion should be anterograde or retrograde, unilateral (through the right carotid artery) or bilateral (through both carotid arteries), most guidelines recommend anterograde cerebral perfusion (ACP) through the right axillary artery (RAA) during aortic arch surgery [1, 2]. To avoid complications associated with additional infraclavicular incisions and axillary artery cannulation, cannulation of the innominate artery (IA) using a single sternal approach has been increasing in recent years. Here, we performed direct IA cannulation using a pediatric aortic cannula and sought to evaluate the safety and efficacy of this method in patients with acute Stanford type A aortic dissections (ATAAD) who underwent total arch replacement (TAR) with the frozen elephant trunk (FET) technique.

## Methods

### Patients

Between October 2020 and November 2021, 29 patients with ATAAD underwent TAR using the FET technique under moderate hypothermic circulatory arrest (HCA) with ACP established by direct IA cannulation. Data were collected from medical records. This retrospective study was approved by the institutional review board, and all patients provided signed informed consent before undergoing surgery.

### Surgical technique

In addition to standard anesthesia monitoring, bilateral radial arterial lines were used. Cerebral perfusion was monitored using near-infrared spectroscopy (Somanetics, Covidien, Mansfield, MA, USA). All patients underwent full median sternotomy. The innominate vein was isolated and pulled caudally before pericardiotomy. The three major neck vessels were identified. The base of the IA was then dissected and the umbilical cord was wrapped around it (Fig. 2A). A 5-0 Prolene purse-string suture was then applied to the anterior surface of the IA approximately 2 cm distal to its origin.

**Fig. 1:**
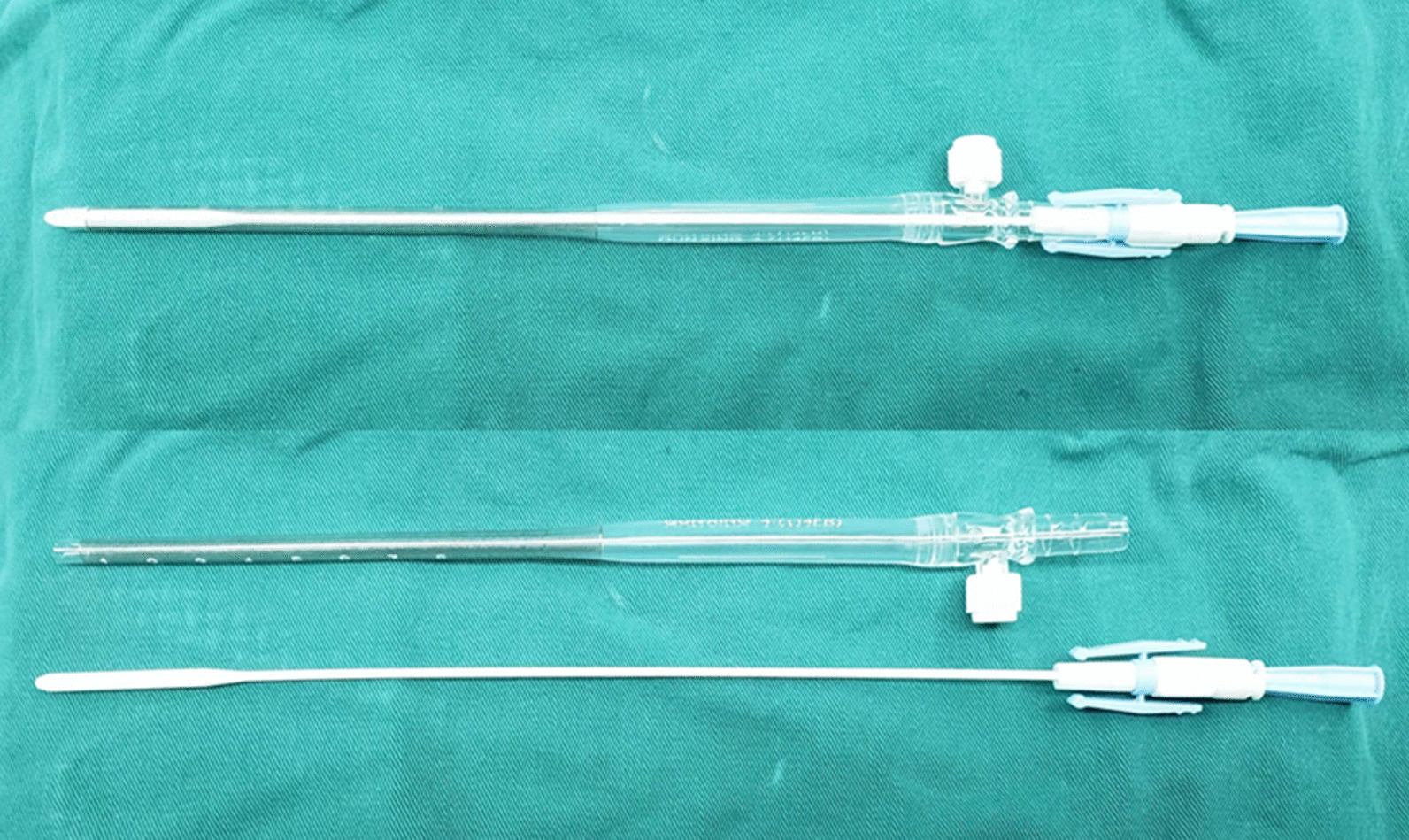
The 14F pediatric arterial cannula used for cannulating the innominate artery to enable antegrade cerebral perfusion

**Fig. 2:**
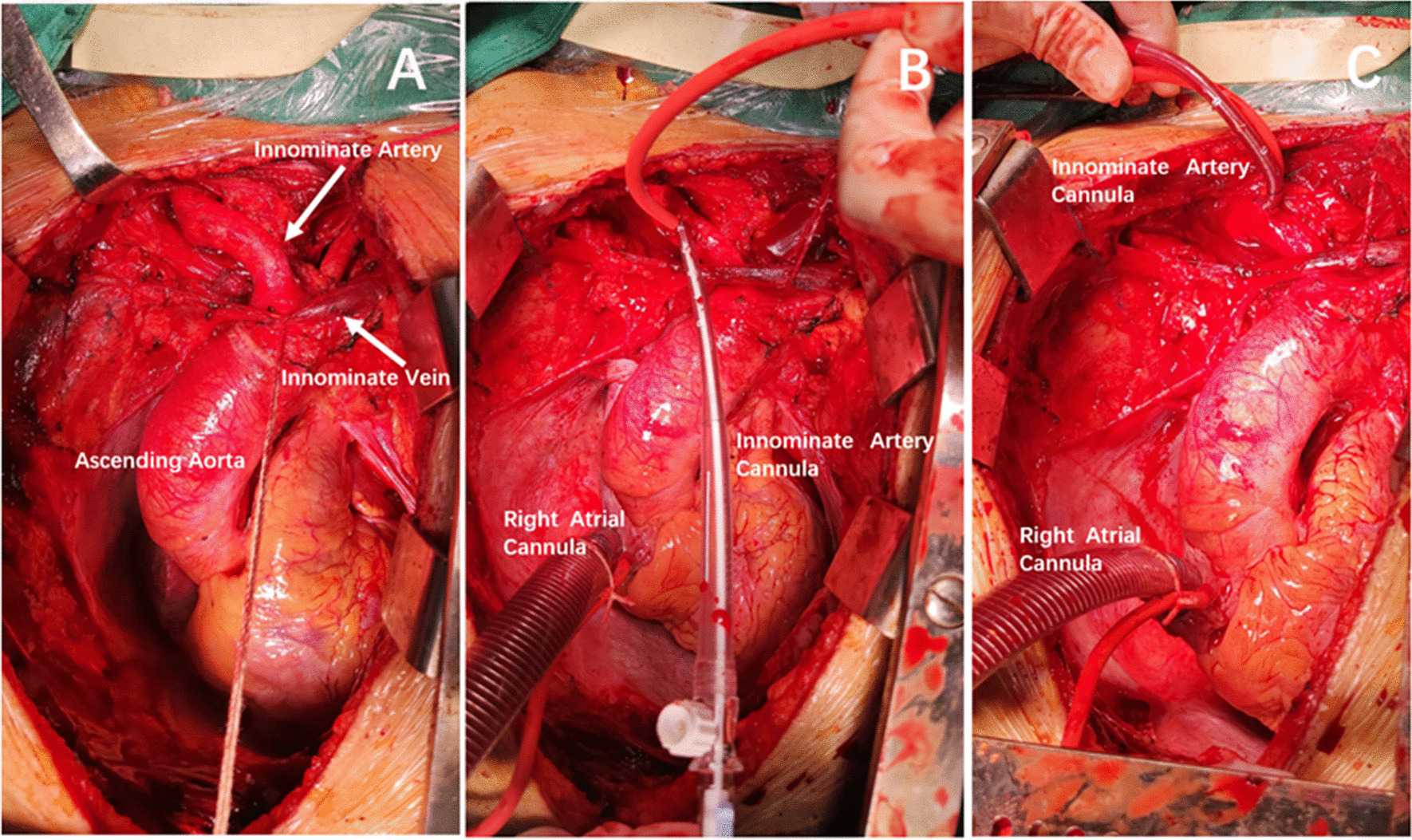
**A** The innominate vein was isolated and pulled caudally and the innominate artery (IA) was dissected. **B** A 14F or 16F pediatric arterial cannula was inserted directly into the IA in the direction of the right common carotid artery and secured with purse-string sutures. **C** The cannula was placed in the vessel at a depth of 4 cm to ensure that the proximal end was inserted into the right common carotid artery

After systemic heparinization was achieved, a hole was made at the center of the purse-string suture with the scalp. A 14F or 16F pediatric arterial cannula (Kangxin Medical Instruments Co., Ltd., Changzhou, China) was inserted directly into the IA in the direction of the right common carotid artery and secured with purse-string sutures (Figs. 1, 2B). The cannula was placed in the vessel at a depth of 4 cm to ensure that the distal end was inserted into the right common carotid artery. The 3/8, 3/8, and 1/4-Y connectors were used as the arterial lines for the cardiopulmonary bypass (CPB). The 1/4 connector was connected to the IA cannula, and the 3/8 connector was connected to the femoral artery cannula. A two-stage right atrial cannula was placed in the right atrium (Fig. 2C). CPB was then initiated. Systemic cooling was initiated to bring the bladder temperature to 23–26 °C. Left ventricular venting was initiated via the right superior pulmonary vein and directed towards the left ventricle through the mitral valve. The aorta was clamped, and the heart was arrested in the usual manner.

Proximal aortic surgery was performed while the patient was undergoing cooling. After the target core temperature was reached, the CPB perfusion flow was reduced, the IA, left common carotid, and left subclavian arteries were clamped and severed, and the femoral artery cannula was clamped. During circulatory arrest, ACP was initiated through a pediatric aortic cannula at a flow rate of 5–20 mL/kg/min. Adjustments were made based on radial artery pressure and continuous non-invasive monitoring of cerebral oxygen saturation using near-infrared spectroscopy. Right radial pressure was maintained at 40–60 mm Hg during the HCA. If the left cerebral blood oxygen measurement decreased by 20% of the baseline value, a balloon-tipped cannula was inserted into the left carotid artery and bilateral cerebral perfusion was performed during circulatory arrest. However, this was not observed in this group of patients. The diseased ascending aorta and aortic arch were resected, the aortic arch was severed between the left subclavian artery and the left common carotid artery, and the FET (Cronus®, MicroPort Medical, Shanghai, China) was implanted into the true lumen of the descending aorta. A four-branch vascular graft (InterVascular SAS, LaCiotat, France) was used to complete the TAR, and one branch of the four-branch vascular graft was used to start CPB after completion of the distal aortic anastomosis. The corresponding artificially branched vessel was anastomosed with the left common carotid artery, after which systemic rewarming was started and the brain was perfused bilaterally, followed by left subclavian artery reconstruction and proximal aortic anastomosis. Myocardial perfusion was resumed, and the IA was anastomosed. The pediatric arterial cannula was removed after IA reconstruction.

## Results

The preoperative statistical data of the patients are shown in Table 1. The mean patient age was 50.9 ± 9.47 years, and 82.8% of them were male. Table 2 describes the details of the aortic surgeries. Aortic root plasty was performed in 27 patients, while the Bentall procedure was performed in two patients. The mean CPB, aortic cross-clamp, and ACP times were 202 ± 29, 141 ± 28, and 25 ± 6.4 min, respectively. The lowest mean core temperature was 23.8 ± 0.74 °C. The mean cerebral blood flow was 5–20 mL/kg/min through 14F or 16F arterial cannulation. No complications were associated with the IA cannulation. The perioperative results are presented in Table 3. The perioperative mortality rate was 3.4%. The median ICU stay was 129 ± 59 h. Perioperative stroke occurred in two cases (6.9%). Postoperative delirium was observed in four patients (13.8%), but brain computed tomography (CT) showed no neurological injury, and all patients were discharged without symptoms.

**Table 1:** Preoperative patient characteristics

Variable	Value
*Characteristics*	
Age, years (mean ± SD)	50.9 ± 9.47
Male sex, n (%)	24 (82.8)
BMI, kg/m^2^ (mean ± SD)	26.5 ± 3.48
Smoker, n (%)	13 (44.8)
Hypertension, n (%)	25 (86.2)
Family history of heart or aortic disease, n (%)	1 (3.4)
Previous myocardial infarction, n (%)	2 (6.9)
Largest aortic diameter by CT, mm (mean ± SD)	45.0 ± 7.01
Recent stroke, n (%)	2 (6.9)
Chronic lung disease, n (%)	3 (10.3)
Renal failure, n (%)	1 (3.4)
Marfan syndrome, n (%)	1 (3.4)

Data are presented as mean ± standard deviation or n (%)

BMI, body mass index; CT, computed tomography; SD, standard deviation

**Table 2:** Operative data

Variable	Value
*Operative data*	
Proximal aortic reconstruction	
Aortic root plasty, n (%)	27 (93.1)
Bentall procedure, n (%)	2 (6.9)
Concomitant procedures	
CABG, n (%)	2 (6.9)
CPB time, min (mean ± SD)	202 ± 29
Cross-clamp time, min (mean ± SD)	141 ± 28
ACP time, min (mean ± SD)	25 ± 6.4
Lowest nasopharyngeal temperature, °C (mean ± SD)	23.8 ± 0.74

Data are presented as mean ± standard deviation or n(%)

ACP, antegrade cerebral perfusion; CABG, coronary artery bypass grafting; CPB, cardiopulmonary bypass; SD, standard deviation

**Table 3:** Perioperative outcomes

Variable	Value
*Perioperative outcomes*	
Re-exploration for surgical bleeding, n (%)	1 (3.4)
Perioperative myocardial infarction, n (%)	1 (3.4)
New renal failure requiring dialysis, n (%)	0 (0)
Ventilation, h (mean ± SD)	54 ± 35.3
Pulmonary complication, n (%)	11 (37.9)
Tracheostomy, n (%)	0 (0)
ICU stay, h (mean ± SD)	129 ± 59
Stroke, n (%)	2 (6.9)
Delirium, n (%)	4 (13.8)
Multi-system organ failure, n (%)	1 (3.4)
Limb ischemia, n (%)	1 (3.4)
Paraplegia, n (%)	3 (10.3)
Deep sternal wound infection, n (%)	1 (3.4)
30-day/in-hospital mortality, n (%)	1 (3.4)

Data are presented as mean ± standard deviation or n (%)

ICU, intensive care unit; SD, standard deviation

## Discussion

Thoracic aortic surgery has a high incidence of neurological complications. Brain protection strategies have been developed over the years to reduce neurological complications during aortic arch surgery. Deep hypothermic circulatory arrest (DHCA) alone was the initial primary neuroprotective approach, and retrograde cerebral perfusion (RCP) was later introduced to improve brain protection during DHCA. It was not until Sabik et al. performed axillary artery cannulation that ACP became possible [3]. ACP makes the cerebral blood flow more uniform, providing a longer safe circulatory arrest time and better brain protection for complex aortic surgery. ACP allows the use of moderately low temperatures, thereby shortening the cooling and rewarming phases [4, 5]. Perreas et al. demonstrated that ACP reduces neurological complications and mortality after aortic arch surgery [6]. These advantages have made ACP via the RAA cannulation the standard brain protection method in aortic arch surgery [7]. Two different techniques have been described: the side-graft technique and direct cannulation. However, axillary artery cannulation requires an additional infraclavicular incision and increased operative time, which can lead to potential complications including seroma or hematoma formation, brachial plexus injury, limb mal-perfusion, and aortic dissection [8–10]. In recent years, the IA has become an alternative cannulation site [11]. During thoracic aortic surgery, IA cannulation does not require a second incision, reduces surgical time, and is always within the surgeon's view. The data showed no statistically significant difference in the in-hospital mortality rate at the cannulation site between RAA (6%) and IA (5.22%) (*P* = 0.55). There were no significant differences between permanent and temporary nerve defects in RAA and IA cannulations [12].

IA cannulation can be accomplished either directly or by sewing a side graft. Preventza et al. described ACP using an 8-mm Dacron graft sewn to the IA in an end-to-side fashion in 263 aortic surgeries, 33 of which were total arch surgeries, with an overall mortality rate of 4.9% and stroke rate of 3.4% [13]. The same group subsequently published a comparison of 515 patients treated with RAA cannulation and 376 patients treated with IA cannulation (all with side grafts) during elective aortic operations. No intergroup differences were reported in surgical mortality, total stroke, or site-related complications [14]. A similar technique for IA cannulation described by Uchino et al. resulted in a 2.5% stroke rate in 159 aortic surgery patients (14 Stanford type A dissections), including 132 TARs [15]. It is important to note that sewing of the side graft to the IA requires partial occlusion of the artery for anastomosis, which may impair the right cerebral blood flow and cause vascular damage. The advantages of direct IA cannulation are reduced operation time, the ease of operation, and avoiding the need to suture the side graft and its inherent problems. Ji et al. placed a specially designed 22F–24F angle cannula directly into the artery. The cannula tip was oriented toward the aortic arch to allow CPB and then it was rotated toward the head to allow ACP before HCA. Sixty-eight patients who underwent proximal aortic arch surgery were treated with this method. Two patients died, and none experienced neurological complications [16]. Kashani et al. applied a 20F or 22F arterial cannula to direct IA cannulation in 14 cases of ascending aorta or hemiarch replacement. There were no deaths, and two patients had postoperative neurological complications [17]. The IA is generally 10–15 mm in diameter, although no cannulation-related complications were reported in these series, introducing such a large cannula (20F–24F) into the IA may damage the posterior wall of the vessel. In addition, the use of a large cannula requires multiple rotations of the cannula between systemic perfusion and ACP delivery, further increasing the risk of vascular injury. Therefore, many patients with normal-sized IAs may not be candidates for large cannulas, the use of which is limited to those with large-diameter arteries. Jasser et al. cannulated the IA directly with a 9F standard-tip aortic root cannula after hypothermia and circulatory arrest. Among 100 patients who underwent elective hemiarch reconstruction, one operative death and two poor neurological outcomes were reported [18]. Payabyab et al. used a 7F aortic root cannula for IA cannulation in 75 Stanford Type A dissection surgeries (including seven TARs). The incidence of perioperative stroke was 9.3%, and the 30-day mortality rate was 14.7% [19]. However, achieving adequate cerebral perfusion using a small-caliber cannula is challenging in larger patients. In 50 cases of ascending aorta or hemiarch replacement, Garg et al. inserted a 14F pediatric venous cannula into the IA through a 0.035-in. J-tipped guidewire. This diameter was sufficient to achieve the desired perfusion pressure and flow rate (> 1 L/min). The operative mortality rate was 2% [20]. Sang et al. used the modified Seldinger technique to place a flexible 12F or 14F pediatric arterial cannula (Medtronic Biomedical, Inc.) through a guidewire into the IA without pre-dilation. Ascending aorta or hemiarch replacement was performed in 42 aortic aneurysms, with a perioperative mortality of 2% and no cases of stroke [21]. Blind guidewire placement or the repeated use of dilators in the IA can increase the risk of stroke.

We used 14F or 16F flexible pediatric arterial cannulas for direct placement in the IA. Pediatric arterial cannulas are not limited by IA size and can be widely used in patients with different body sizes. Instead of a taper-tipped cannula inserted through a guidewire as described in other reports, we chose a blunt-tipped cannula to avoid possible damage to the posterior wall of the IA. Unlike the 1–1.5 cm intravascular depth reported by Wai Sang, we used a depth of 4 cm to ensure the correct insertion of the cannula tip into the right common carotid artery and to avoid cannulation sliding into the right subclavian artery. In addition, our method avoids any interruption of cerebral blood flow and eliminates surgical field clutter compared with open cannulation through the IA ostium, which is typically performed with a balloon-tipped cannula. With technological advancements, TAR using a four-branch graft and a stented elephant trunk has been widely used in ATAAD achieving satisfactory long-term clinical results [11, 22, 23]. We used double arterial cannulation (femoral and innominate arteries) to achieve better systemic perfusion and systemic cooling. Our study has some limitations. Direct IA cannulation is not recommended in patients undergoing a second surgery, IA dissection, or calcification. If the femoral artery was not suitable for CPB, double arterial cannulation was abandoned, and an 8 mm Dacron Graf was sewn end-to-side to the non-dissected IA to achieve cannulation for CPB. Previous reports suggested that dissection may involve IA, and direct IA cannulation is not recommended for ATAAD patients undergoing total arch surgery [20, 21]. However, our experience suggests that IA dissection occurs only in some patients with ATAAD. Preoperative imaging evaluation was performed on 44 ATAAD patients (including 29 patients of this study) who underwent TAR during the same period in our center; of them, an obvious IA dissection was found in 15 patients, all of whom underwent RAA cannulation. Among these 15 patients, five died during the perioperative period. We believe that the main reason for this is that patients underwent RAA cannulation have a wider range of dissection and are in a more critical condition.

## Conclusion

Compared with other ACP methods, direct IA cannulation using a pediatric arterial cannula is a simple, safe, and rapid technique for establishing ACP during TAR with the FET technique for ATAAD. The contraindication for this technique is arterial dissection in the proximal IA, which should be evaluated by preoperatively contrast-enhanced CT, and careful intraoperative exploration. This single-center study had several limitations, including its observational rather than comparative design and small sample size. Validation in larger samples will be required.

## Data Availability

All data generated or analyzed, as well as the materials used during this study, are included within the article.

## Abbreviations

ATAAD: Acute Stanford type A aortic dissections
FET: Frozen elephant trunk
ACP: Antegrade cerebral perfusion
IA: Innominate artery
TAR: Total arch replacement
HCA: Hypothermic circulatory arrest
CPB: Cardiopulmonary bypass
CT: Computed tomography
DHCA: Deep hypothermic circulatory arrest
